# The Phenotypic Effects of Royal Jelly on Wild-Type *D*. *melanogaster* Are Strain-Specific

**DOI:** 10.1371/journal.pone.0159456

**Published:** 2016-08-03

**Authors:** Stefanie L. Morgan, Joseph A. Seggio, Nara F. Nascimento, Dana D. Huh, Jasmin A. Hicks, Katherine A. Sharp, Jeffrey D. Axelrod, Kevin C. Wang

**Affiliations:** 1 Department of Dermatology, Program in Epithelial Biology, Stanford University School of Medicine, Stanford, CA, 94305, United States of America; 2 Program in Cancer Biology, Stanford University School of Medicine, Stanford, CA, 94305, United States of America; 3 Department of Biological Sciences, Bridgewater State University, Bridgewater, MA, 02325, United States of America; 4 Department of Pathology, Stanford University School of Medicine, Stanford, CA, 94305, United States of America; 5 Department of Genetics, Stanford University School of Medicine, Stanford, CA, 94305, United States of America; 6 Veterans Affairs Healthcare System, Palo Alto, CA, 94304, United States of America; University of Mississippi, UNITED STATES

## Abstract

The role for royal jelly (RJ) in promoting caste differentiation of honeybee larvae into queens rather than workers is well characterized. A recent study demonstrated that this poorly understood complex nutrition drives strikingly similar phenotypic effects in *Drosophila melanogaster*, such as increased body size and reduced developmental time, making possible the use of *D*. *melanogaster* as a model system for the genetic analysis of the cellular mechanisms underlying RJ and caste differentiation. We demonstrate here that RJ increases the body size of some wild-type strains of *D*. *melanogaster* but not others, and report significant delays in developmental time in all flies reared on RJ. These findings suggest that cryptic genetic variation may be a factor in the *D*. *melanogaster* response to RJ, and should be considered when attempting to elucidate response mechanisms to environmental changes in non-honeybee species.

## Introduction

In the social honeybee *Apis mellifera*, the royal jelly (RJ) diet is known to be responsible for the development of female larvae into queen rather than worker bees[1–3]. Fertile queen bees are markedly larger, develop more rapidly, and have significantly longer lifespans than sterile worker bees[1–3]. Interestingly, differentiation of honeybee larvae into queens rather than workers is not the result of underlying differences in genotype, but instead has been shown to be a direct consequence of epigenetic changes brought about through larval ingestion of RJ[4,5]. These effects of RJ were previously shown to be conserved in *Drosophila melanogaster*, as rearing *D*. *melanogaster* in the presence of RJ decreased developmental time, increased body size, and increased fertility and lifespan of the female progeny[6]. It was concluded from these findings that RJ acts across insect species, implying a functionally conserved mechanism of action.

It is interesting to note, however, that this previous study demonstrated phenotypic and physiological effects of RJ using only one wild-type *D*. *melanogaster* strain, *Canton-S* (*Can-S*). Multiple wild-type strains of *D*. *melanogaster* exist, and significant differences in responses to allelic mutations and environmental stimuli have been observed both between and even within certain wild-type strains[7–9]. As a result, wild-type organisms utilized in a study must be strictly controlled to avoid confounding experimental variables, however, doing so often provides a limited view of what occurs in nature, as the alleles in a genome usually vary among individuals[10–13]. Furthermore, cryptic genetic variation (CGV), which does not contribute to the normal range of phenotypes observed in a population, can significantly modify an existing phenotype in response to environmental changes[13]. Such a complex phenomenon could be particularly important to consider when introducing an abnormal food source, such as RJ, into a *D*. *melanogaster* population.

The importance of CGV and wild-type strain differences became apparent when we attempted to replicate previously reported RJ-fed *D*. *melanogaster* findings[6]. Despite multiple attempts, we were only able to replicate increases in the size of RJ-fed flies when using the *Can-S D*. *melanogaster* strain. Furthermore, in contrast to a previous report[6], we observed increased developmental times for every *D*. *melanogaster* strain reared on RJ containing food. Our findings suggest that the differential responses to RJ may be the result of CGV, and reaffirm the importance of considering genetic background dependence as a critical determinant capable of modifying phenotypes and/or behaviors that arise after environmental challenges.

## Materials and Methods

*Canton-S* and *Oregon-R Drosophila melanogaster* were procured from Bloomington (stocks #1 and #5, respectively), and were maintained as isogenized laboratory strains. All flies were kept in an incubator with a 12:12 LD cycle at a temperature of 23–25.5°C, with an unregulated humidity ranging from 50–60%. Both strains were maintained on 2.5g of instant fly food (proprietary Carolina Formula 4–24 Medium, Blue; Burlington, NC) combined with 10 mL of water. Pure honeybee royal jelly (RJ) was a gift from Dr. Ryszard Maleszka, which he procured from his own laboratory’s colonies. It was stored at 4°C and protected from light when not in active use.

Fly food for the rearing experiments was prepared by adding RJ to instant fly food through manual mixing of the appropriate amount of RJ (0%, 10%, 15%, 20%, or 25% as indicated, w/v) into 10 ml of water prior to addition of the water to 2.5g instant food.

To assess differences in body size in both strains of *D*. *melanogaster* in response to RJ, mating was conducted by initially placing five male and five female flies into a vial, and upon confirmation of larvae visibly present within the vial, removing parents. Vials were visually checked daily, and action was taken for each vial as needed. Eclosed flies were removed from the vial after each eclosion period, and whole body length of male and non-virgin female flies was subsequently measured using standard millimeter micro-calipers under a dissecting microscope. To ensure greater accuracy of measurements, females were not measured until they had completely shed their pupal waste products, but before they any signs of a distended abdomen. Measurements were made across multiple vials for each condition (of RJ%), until a sufficient number of flies had been measured for each condition.

Pilot experiments were conducted at Stanford University and replicated at Bridgewater State University, however, finding of no difference between initial preliminary results prompted collaborative data collection between the two universities thereafter. Data from both locations was gathered in an identical fashion and was combined for all analyses after no difference was observed between data collected at either location (as described below).

Length of time until the first eclosion was determined for at least two vials for both strains across all conditions (of RJ%) by visual inspection of the vials after removal of the parental generation (mating and parental generation removal as described above). Vials were visually inspected every dusk (1 hr before lights off) until flies began to eclose, and then continued to be inspected visually in this way for three consecutive days thereafter.

Statistical analyses were performed using one-way ANOVA, with Tukey HSD for subsequent pairwise comparisons (or Dunnett’s T3 for comparisons with unequal variances found by a Levene’s Test), for both time to first eclosion and body length in male and female RJ fed flies compared to control fed flies. Data obtained from both Stanford University and Bridgewater State University were combined for these analyses, as a Two-Way ANOVA revealed no significant difference between the results obtained in any experiments at the two different institutions (F_1,279_ = 0.13, *p* = 0.72).

## Results

**Fig 1:**
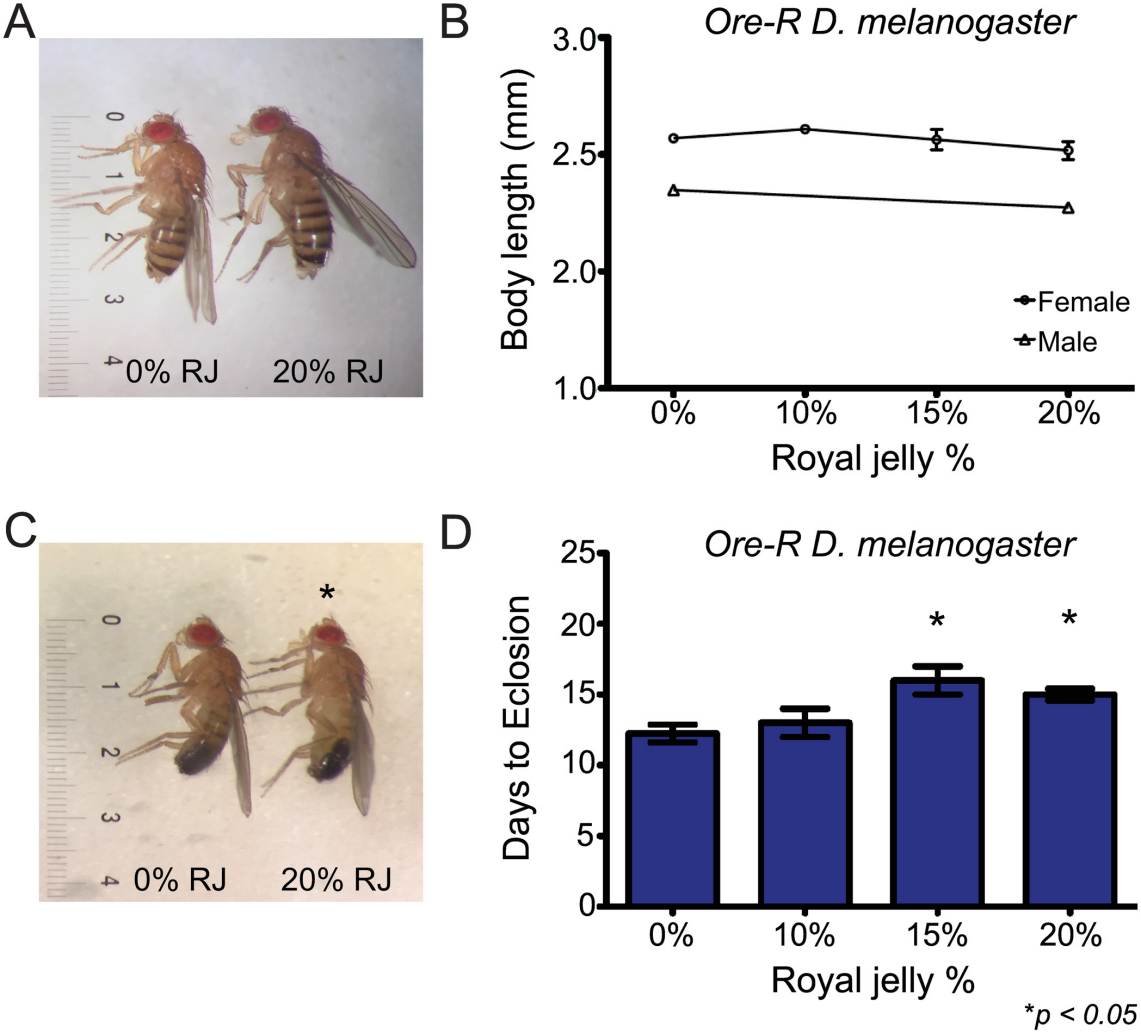
RJ reduces body size of male but not female *Ore-R D*. *melanogaster*, and increases developmental time for both sexes in a dose-dependent manner. (A, B) Female *Ore-R* flies reared on food containing 10% (n = 72), 15% (n = 24), and 20% (n = 24) RJ demonstrated no differences in size compared to controls (n = 87; F_2,180_ = 2.80, *p* = 0.063, Levene’s *p*<0.001, all subsequent pairwise comparisons *p*>0.10), while (B, C) males reared on 20% RJ (n = 41) were slightly smaller than controls (n = 44; t_1,83_ = 3.56, *p* = 0.001). (D) However, increasing amounts of RJ significantly delayed larval development times for both male and female flies raised on 15% and 20% RJ in multiple vials (n ≥ 2 vials for each condition; F_3,8_ = 6.49, *p* = 0.015). Ruler lines indicate 0.1mm intervals. *denotes significant difference relative to control at a value of at least *p < 0*.*05*.

We were interested in reproducing previous findings that rearing *Drosophila melanogaster* on RJ resulted in larger female flies with a shortened developmental time[6]. We initially tested the effects of RJ on *D*. *melanogaster* development utilizing *Oregon-R D*. *melanogaster* (*Ore-R*). However, when female flies were reared on food containing 10% (n = 72), 15% (n = 24), and 20% (n = 24) RJ, there was no significant difference in body length of the progeny compared to controls (n = 87) reared on 0% RJ (F_2,180_ = 2.80, *p* = 0.063, Levene’s *p*<0.001, all subsequent pairwise comparisons *p*>0.10; Fig 1A and 1B, S1A Fig and S1 Data). Additionally, we observed that in *Ore-R* males, flies were smaller when raised on 20% RJ (n = 41) compared to 0% RJ (n = 44; t_1,83_ = 3.56, *p* = 0.001; Fig 1B and 1C, S1B Fig, S1 Data). Furthermore, in a subset of 0% (n = 4), 10% (n = 2), 15% (n = 2), and 20% (n = 4) vials selected for additional time to eclosion analysis, increasing percentages of RJ in the food resulted in significant delays in larval developmental timing in terms of time to first eclosion (F_3,8_ = 6.49, *p* = 0.015; Fig 1D and S2 Data) for both 15% *p* = 0.024) and 20% (*p* = 0.040) groups.

**Fig 2:**
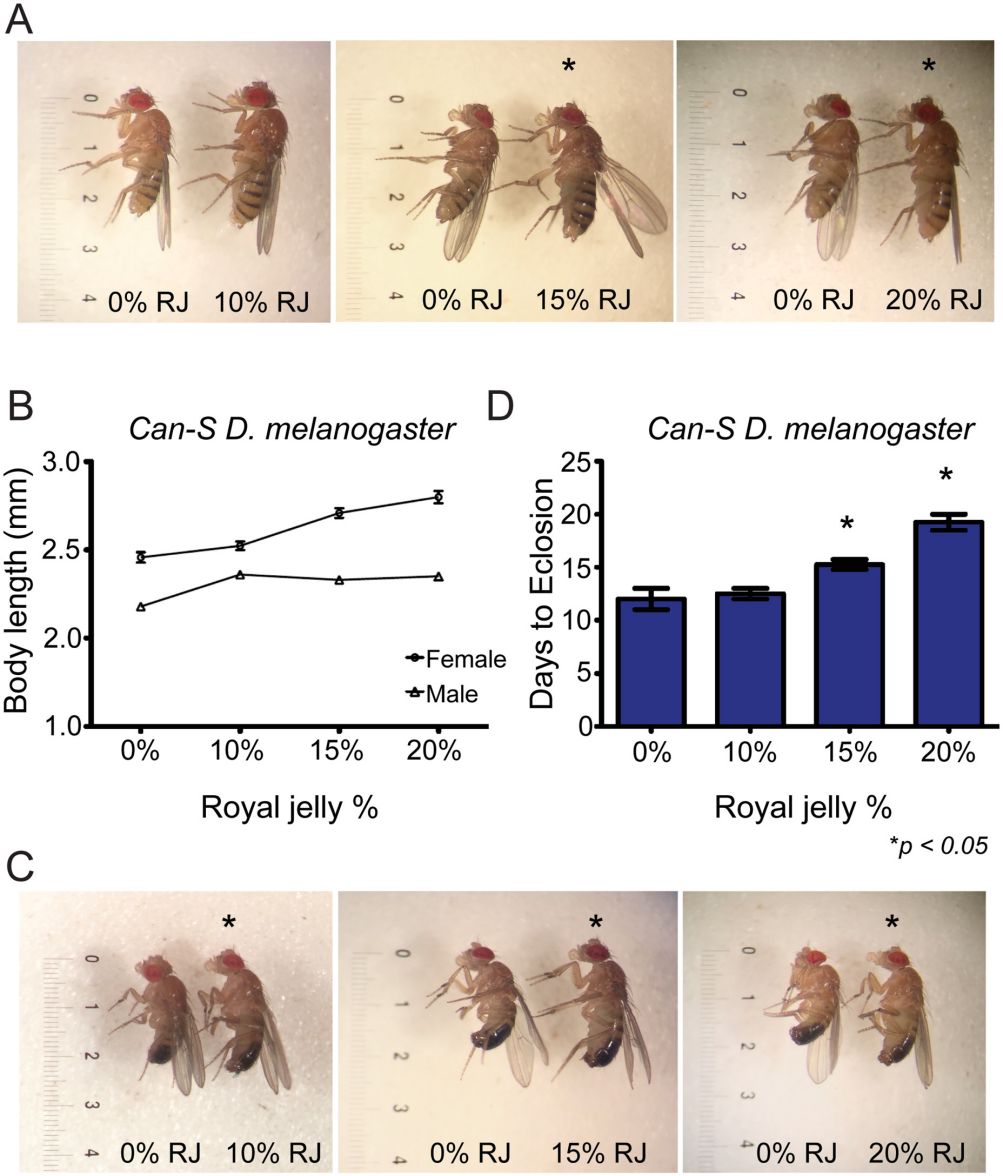
RJ increases body size of *Can-S D*. *melanogaster* and extends developmental time in a dose-dependent manner. (A, B) Female *Can-S* flies reared on 15% (n = 52) or 20% (n = 34) RJ but not those reared on 10% (n = 63) were significantly longer than controls (n = 65; F_3,210_ = 26.63, *p*<0.001, Levene’s *p* = 0.12, *p*<0.001). Further, while body length increased in a dose-dependent manner, and flies reared on 10% RJ were significantly smaller than those reared on 15% (*p* = 0.001) or 20% RJ (*p*<0.001) RJ, flies reared on 15% RJ were not significantly smaller than those reared on 20% RJ (*p* = 0.33). (B, C) Similarly, male *Can-S* flies reared on 10% (n = 45), 15% (n = 20), or 20% (n = 6) RJ were also significantly larger than controls (n = 29; F_3,96_ = 30.27, *p*<0.001, Levene’s *p*<0.001; all pairwise *p*<0.001). However, while male *Can-S* flies raised on 10% RJ were significantly larger than flies raised with 0% RJ, those reared on 15% and 20% RJ did not significantly differ from flies raised on 10% RJ (all *p*>0.75). (D) Increasing amounts of RJ significantly delayed larval development times in multiple vials of flies raised on 15% and 20% RJ (n = 4 for all; *p* = 0.009). *: significance at *p* < 0.01. Ruler lines indicate 0.1mm intervals. *denotes significant difference relative to control at a value of at least *p < 0*.*05*.

As a prior study reporting larger *D*. *melanogaster* size was conducted with *Canton-S D*. *melanogaster* (*Can-S*)[6], we hypothesized that the dramatic phenotypic effects of RJ could be a manifestation of CGV. To test this, we repeated our RJ experiments using *Can-S* flies. Interestingly, we found that female *Can-S* flies reared on 15% (n = 52) and 20% (n = 34) RJ exhibited a significantly longer body length compared to controls (n = 65), and that body length increased in a dose-dependent manner (F_3,210_ = 26.63, *p*<0.001, Levene’s *p* = 0.12, *p*<0.001 for 15% and 20% comparisons to 0%; Fig 2A and 2B, S1C Fig, and S1 Data). No change in body length was observed for female flies reared on 10% RJ (n = 63) compared to controls. Subsequent pairwise comparisons showed that of the female *Can-S* flies, those reared on 10% RJ were significantly smaller than those reared on 15% (*p* = 0.001) or 20% RJ (*p*<0.001), but that flies reared on 15% RJ were not significantly smaller than those reared on 20% RJ (*p* = 0.33; Fig 2A and 2B).

For male *Can-S* flies, slight size differences were observed when they were raised on 10% (n = 45), 15% (n = 20), or 20% (n = 6) RJ compared to controls (n = 29; F_3,96_ = 30.27, *p*<0.001, Levene’s *p*<0.001; all pairwise *p*<0.001, Fig 2B and 2C); however, no size differences were observed in flies raised on 10% RJ or above (all *p*>0.75). However, we observed much fewer live male flies in higher percentages of RJ compared to flies raised on 10% RJ (n = 45), possibly indicating that male *Can-S* flies are more sensitive to RJ than female flies. As with the *Ore-R* flies, we selected a subset of 0%, 10%, 15%, and 20% RJ (all n = 4) *Can-S* vials for more in-depth time-to-eclosion analysis. We observed that increasing the percentages of RJ in the food to 15% or 20% correlated with marked delays in larval development (F_3,12_ = 21.63, *p*<0.001) in a dose dependent manner (*p* = 0.033 and *p*<0.001 for 15% and 20% respectively; Fig 2D and S2 Data). Additionally, of the *Can-S* flies, those raised on 20% RJ took significantly longer to eclose compared to those on 15% RJ (*p* = 0.009), and flies reared on 15% RJ took marginally longer than 10% RJ flies (*p* = 0.076). Flies raised with concentrations of RJ above 20% exhibited no eclosions.

## Discussion

Our findings demonstrate that many of RJ’s effects on honeybees appear to be preserved in *Drosophila melanogaster*, but only in some strains. This is consistent with a large body of evidence indicating that CGV can have significant experimental impact[7–9,14–18], and highlights how profoundly genetic background can influence phenotypic outcomes. As there is evidence that RJ differentially effects longevity, body size, and fecundity, of multiple other species, such as silkmoths[19], crickets[19], *C*. *elegans*[20], and mice[21], it will be important to consider how CGV may be influencing such observations. This will be particularly true for studies aiming to elucidate the mechanism by which RJ response has been retained across species. For example, as the EGFR signaling pathway has been specifically implicated in RJ response[6], observations that the phenotypic effects of EGFR knockout mice were genetic background dependent[18] will be important to take into consideration when investigating RJ response in mammals. Taken together, this emphasizes the importance of considering CGV in studies seeking to elucidate RJ’s effects in non-honeybee species.

In contrast to our findings of body size differences in *D*. *melanogaster*, we failed to validate a previous report that RJ reduces *D*. *melanogaster* developmental time[6]. Rather, we consistently observed significant dose-dependent developmental delays in both *D*. *melanogaster* lines reared in the presence of RJ, a finding which was substantiated in work from Shorter et.al[22] published while this manuscript was under preparation. Further, we also failed to replicate a previous report of female-specific RJ effects in *Can-S D. melanogaster[6]*, instead repeatedly finding that it increased body size of both sexes in this strain. Shorter et al.[22] confirmed this finding as well, not only in *wt1118 Can-S B D*. *melanogaster*, but also in three additional wild-type lines they collected[22]. In further keeping with our results, Shorter et al. similarly reported strain-specific differences in RJ response, finding that RJ decreased fly size in one of the four lines they observed. Though a number of experimental controls would be required to make the two studies fully comparable, our and Shorter et al.’s work demonstrate strain-specific responses to RJ, and highlight the importance of accounting for CGV in elucidating RJ’s mechanism of function in species other than the honeybee.

Taken together, it is clear that the impacts of CGV on RJ studies should not be taken lightly. Although we investigated differential effects of RJ in only two wild-type strains of *D*. *melanogaster* in this work, it is likely that extending this study to a greater number of wild-type strains would uncover a number of additional phenotypic differences that could lend greater insight into RJ’s mechanism of action. In summary, our findings indicate that obtaining a comprehensive understanding of RJ’s effects will require a multidimensional approach that takes into account the diverse effects that standing genetic variations can produce.

## Supporting Information

S1 Data Effects of RJ on *D*. *melanogaster* body length. (XLSX)

S2 Data Effects of RJ on time to first eclosion. (XLSX)

S1 Fig RJ effects body size of only *Can-S* flies. Additional photographs of *Ore-R* and *Can-S* flies confirms that (A) female *Ore-R* flies reared on food containing 20% RJ demonstrated no differences in size compared to controls while (B) male *Ore-R* flies reared on 20% RJ were slightly smaller than controls. In contrast, both (C) female and (D) male *Can-S* flies raised on 20% RJ were significantly larger than controls. However, a significant change in body length was not observed in (C) female flies raised on 10% RJ.
(TIF)
