# Supplementary figures and images for: The Phenotypic Effects of Royal Jelly on Wild-Type D. melanogaster Are Strain-Specific
Source: PLoS One. 2016 Aug 3;11(8):e0159456. doi: 10.1371/journal.pone.0159456 (PMC4972316; doi:10.1371/journal.pone.0159456)

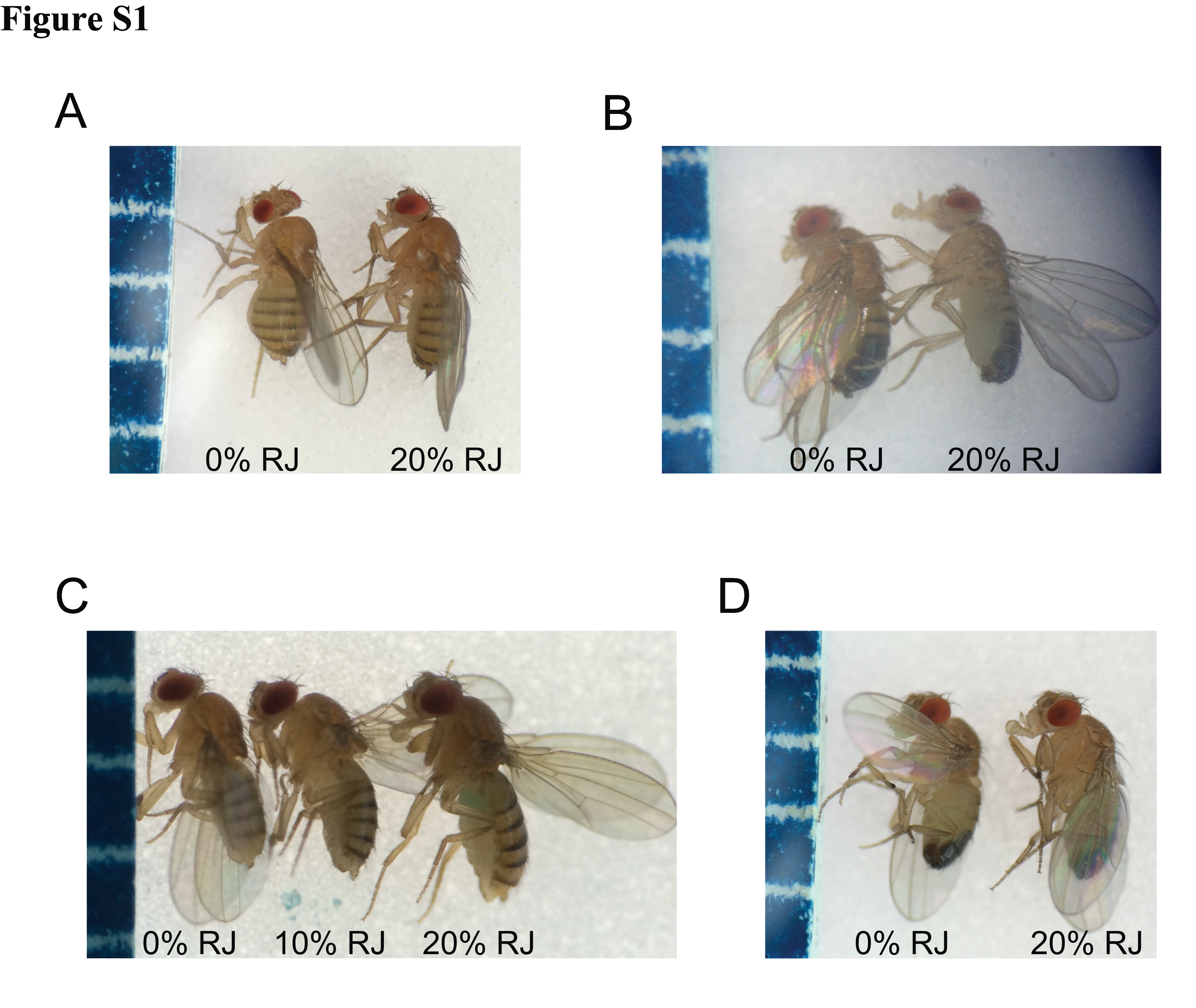

Supplement: S1 Fig — Additional photographs of Ore-R and Can-S flies confirms that (A) female Ore-R flies reared on food containing 20% RJ demonstrated no differences in size compared to controls while (B) male Ore-R flies reared on 20% RJ were slightly smaller than controls. In contrast, both (C) female and (D) male Can-S flies raised on 20% RJ were significantly larger than controls. However, a significant change in body length was not observed in (C) female flies raised on 10% RJ. (TIF) [file pone.0159456.s003.tif]
